# Improving HCC Prognostic Models after Liver Resection by AI-Extracted Tissue Fiber Framework Analytics

**DOI:** 10.3390/cancers16010106

**Published:** 2023-12-24

**Authors:** Rokas Stulpinas, Mindaugas Morkunas, Allan Rasmusson, Julius Drachneris, Renaldas Augulis, Aiste Gulla, Kestutis Strupas, Arvydas Laurinavicius

**Affiliations:** 1 Faculty of Medicine, Institute of Biomedical Sciences, Department of Pathology and Forensic Medicine, Vilnius University, 03101 Vilnius, Lithuaniaarvydas.laurinavicius@vpc.lt (A.L.); 2 National Center of Pathology, Affiliate of Vilnius University Hospital Santaros Klinikos, 08406 Vilnius, Lithuania; mindaugas.morkunas@santa.lt; 3 Vilnius Santaros Klinikos Biobank, Vilnius University Hospital Santaros Klinikos, 08661 Vilnius, Lithuania; 4 Faculty of Medicine, Institute of Clinical Medicine, Vilnius University, 03101 Vilnius, Lithuania; 5 Faculty of Medicine, Centre for Visceral Medicine and Translational Research, Vilnius University, 03101 Vilnius, Lithuania; 6 Center of Abdominal Surgery, Vilnius University Hospital Santaros Klinikos, 08410 Vilnius, Lithuania

**Keywords:** hepatocellular carcinoma, liver, hexagonal grid, artificial intelligence, CNN, prognostic modelling, digital pathology, overall survival

## Abstract

**Simple Summary:**

Prognosis of patients after surgical resection for hepatocellular carcinoma (HCC) is often obscured by the variable impact of the tumor and remaining liver properties. We applied a convolutional neural network and hexagonal grid analytics to extract prognostic indicators from collagen microarchitecture within the tumor and adjacent liver tissue. By associating the extracted features from two distinct fiber types with clinical outcomes, we have developed two computational models to predict the overall survival of the patients. We report the independent prognostic roles for reticulin in HCC and fibrillary collagen in the peritumoral liver, highlighting the significance of assessing region-specific and type-specific fiber features. Our study provides evidence that the predictive power of prognostic models of HCC can be enhanced by artificial intelligence solutions generating computational image-based biomarkers.

**Abstract:**

Despite advances in diagnostic and treatment technologies, predicting outcomes of patients with hepatocellular carcinoma (HCC) remains a challenge. Prognostic models are further obscured by the variable impact of the tumor properties and the remaining liver parenchyma, often affected by cirrhosis or non-alcoholic fatty liver disease that tend to precede HCC. This study investigated the prognostic value of reticulin and collagen microarchitecture in liver resection samples. We analyzed 105 scanned tissue sections that were stained using a Gordon and Sweet’s silver impregnation protocol combined with Picric Acid–Sirius Red. A convolutional neural network was utilized to segment the red-staining collagen and black linear reticulin strands, generating a detailed map of the fiber structure within the HCC and adjacent liver tissue. Subsequent hexagonal grid subsampling coupled with automated epithelial edge detection and computational fiber morphometry provided the foundation for region-specific tissue analysis. Two penalized Cox regression models using LASSO achieved a concordance index (C-index) greater than 0.7. These models incorporated variables such as patient age, tumor multifocality, and fiber-derived features from the epithelial edge in both the tumor and liver compartments. The prognostic value at the tumor edge was derived from the reticulin structure, while collagen characteristics were significant at the epithelial edge of peritumoral liver. The prognostic performance of these models was superior to models solely reliant on conventional clinicopathologic parameters, highlighting the utility of AI-extracted microarchitectural features for the management of HCC.

## 1. Introduction

Hepatocellular carcinomas (HCC) constitute up to 80% of all primary liver cancers [1]. Liver cirrhosis—often induced by viral hepatitis B and C, alcohol consumption, chemical toxins, or metabolic liver diseases—precedes HCC in at least 80% of cases [2]. Furthermore, the global rise of obesity and type 2 diabetes leads to the increased incidence of non-alcoholic fatty liver disease (NAFLD), a primary cause of HCC in the absence of cirrhosis [3]. In recent decades, the impact of HCC has grown significantly, now ranking as the third leading cause of cancer-related mortality worldwide [4]. From 1990 to 2019, there was a staggering 70% increase in HCC incidence, resulting in 480,000 attributable deaths, exacerbated by late diagnoses and advanced disease stages [1,5]. While early detection leads to a five-year survival rate of over 70%, the rate plummets to a mere 18% in later stages [4,6]. These trends highlight the necessity for research into surgical techniques, pharmaceutical options, and prognostic factors to meet the challenge posed by HCC.

Unlike many other cancers, post-resection clinical outcomes of HCC patients do not rely solely on tumor properties and success of its therapy. An important additional determinant is the pathology and functional capacity of the residual liver tissue, underscoring the need for comprehensive assessment of both tumor and non-tumor components [7]. This aspect was taken into account by the Barcelona Clinic Liver Cancer (BCLC) staging and treatment strategy. BLCL points to the limitations of scoring systems for liver failure, like the Child–Pugh system and the model for end-stage liver disease (MELD), which fail to accurately predict the loss of liver function specifically in the context of HCC [8]. To address this, BCLC staging incorporates multiple parameters of tumor burden, liver function, and cancer-related symptoms, as well as alpha-fetoprotein (AFP) levels and the albumin–bilirubin score for liver function assessment [9].

Surgical liver resection provides samples containing HCC and liver parenchyma, as well as their interface. This opens multiple opportunities of tissue pathology methods to assess cellular, molecular, and architectural properties of the disease in the spatial context of the tissue microenvironment. A particular aspect that unifies both tumor and non-tumor tissues is represented by properties of the extracellular matrix (ECM), which has been shown to provide rich and quantifiable data of clinical significance. Tumor associated collagen signatures (TACS) have been conceptualized as computational biomarkers to reflect changes in the organization, alignment, and composition of fibers in the stroma that surround and interact with cancer cells [10]. Originally proposed and demonstrated to have prognostic significance in breast cancer tissue by Keely et al., collagen-derived features were subsequently refined and described in oral, gastric, salivary gland, skin neoplasms and benign fibrous lesions [11,12,13,14]. On the other hand, microarchitectural transformations of liver tissue represent progression of chronic liver disease.

At the molecular level, the ECM is represented by co-polymers comprising various types of collagen, non-collagenous glycoproteins, proteoglycans, and other molecules. These composite biological materials have distinct characteristics, and thus bear similarities with metal alloys, as noted by Bruckner [15]. Arranged into fibrillary structures of the interstitial matrix and the basement membrane—the main components of the ECM—they provide a structural foundation for the liver parenchyma [10]. The molecular complexity of ECM became evident in the 1970s with the discovery that normal liver tissue primarily contains three types of collagen: Type I, Type III, and basement membrane collagens. Type III collagen is the main constituent of reticulin fibers [16]. However, persistent liver injury leads to significant alterations of the composition, orientation, and quantity of all collagen types [17]. As fibrosis progresses, Type I collagen accumulates, eventually becoming predominant in the cirrhotic liver [18]. Despite well-established association between cirrhosis and hepatocarcinogenesis, the exact role of Type I collagen remains unclear: some studies associate it with HCC progression [19], whereas others propose that Type I collagen accumulation may have a beneficial effect by mechanically restraining tumor spread [20]. Conversely, the role of Type III collagen is more established, given that the progressive distortion and dissolution of reticulin framework are histopathological hallmarks of HCC [21]. Therefore, a comprehensive analysis of Type I and Type III (reticulin) collagen properties could provide insights for clinical assessment of patients with HCC.

Currently, histochemical or immunohistochemical methods are used to highlight the different types of collagen [22]. Assessment of the fiber microarchitecture can be performed by visual inspection of the patterns; however, rapid development of computational methods in digital pathology brings novel opportunities for high-capacity quantification of the structural patterns [23]. It has been shown that AI solutions are capable of extracting subvisual features with prognostic relevance from liver tissue [24]. Recently, Patil et al. developed a deep learning model for quantifying reticulin (represented by black, silver-impregnated fibers) in HCC tissue after liver resection [25]. They found that a decreased reticulin proportionate area (RPA) was an independent predictor of metastasis, shorter disease-free survival, and worse overall survival in this study. Similarly, Taylor-Weiner et al. demonstrated the utility of a convolutional neural network (CNN) for Ishak and NASH Clinical Research Network fibrosis scoring in trichrome-stained slides [26]. A recent study suggests that liver pathologists are eager for further development of digital pathology and AI integration [27].

Morkunas et al. proposed a more detailed method for extracting collagen-derived prognostic features; they used a CNN to segment collagen from images of tissue microarrays (TMA) containing Picrosirius Red stained samples of ductal breast carcinoma [28]. From 37 features of fiber morphometry, density, orientation, texture, and fractal characteristics, they found an independent prognostic value of observed heterogeneity of distances between collagen fibers, fiber straightness, and variance of fiber orientation angles to predict patient survival, even though their method was limited to samples of small amount of tumor tissue (TMA cores). On the other hand, full-face surgical resection samples contain large amount of data that are also affected by intratissue heterogeneity, including the malignant and non-malignant components and their interfaces. Therefore, it is essential to properly assess the spatial aspects of pathology features extracted. To tackle this complexity, Plancoulaine et al. proposed a hexagonal tiling approach that allowed quantification of intratumoral heterogeneity of biomarker expression in breast cancer [29]. Building upon this method, a tool for automatic detection of the tumor-stroma interface was further developed by Rasmusson et al. [30]. 

In this study, we explored the predictive value of linear reticulin and thick septal collagen fibers in both HCC and adjacent liver. Utilizing AI for fiber segmentation and tissue classification, followed by hexagonal grid subsampling of the data, we extracted fiber-specific features in the spatial context of the tissue components and their interfaces. Our findings indicate the potential utility of these features to predict overall survival of patients undergoing liver resection for HCC.

## 2. Materials and Methods

### 2.1. Study Design

**Figure 1:**
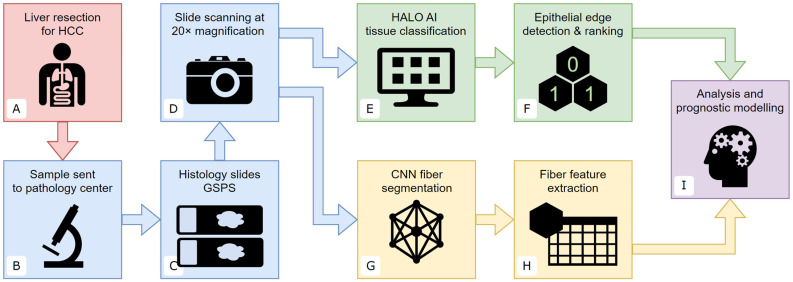
Study design: (**A**) surgical liver resection due to HCC at the Vilnius University hospital Santaros Klinikos (Vilnius, Lithuania); (**B**) specimens tested at the National Center of Pathology (Vilnius, Lithuania); (**C**) samples stained using a modified Gordon and Sweet’s silver impregnation protocol, combined with Picric Acid–Sirius Red; (**D**) slides scanned at 20× magnification (0.5 μm per pixel) using an Aperio^®^ AT2 DX scanner; (**E**) tissue segmentation using HALO^®^ AI on the manual annotations; (**F**) epithelial edge detection and ranking of the hexagonal grid tiles according to tissue class proportions; (**G**) segmentation of reticulin and collagen fibers using a pretrained convolutional neural network, producing an image of red and green fibers against the black background for viewing and analysis; (**H**) calculating pixel-level, fiber-level, and image-level features describing the microarchitecture of the fibers within each hexagon; (**I**) data from individual hexagons are aggregated across predetermined tissue regions to provide case-level features for prognostic modeling.

An overview of the study design is presented in Figure 1. Archived formalin-fixed paraffin-embedded (FFPE) samples from 105 patients were used in this retrospective study. Patients met the following inclusion criteria: (1) they underwent surgical liver resection due to HCC at the Vilnius University hospital Santaros Clinics (Vilnius, Lithuania) with the specimens tested at the National Center of Pathology, an affiliate of the Vilnius University Hospital Santaros Clinics (Vilnius, Lithuania) between 2007 and 2020; and (2) they had at least one archived FFPE block containing both non-necrotic HCC and peritumoral liver tissues. Overall survival (OS) data were obtained with a median OS duration of 938 days and 60% of the individuals deceased as of 2022. The study was approved by the Vilnius Regional Biomedical Research Ethics Committee (permit number 2021/6-1354-843), who waived the requirement of individual informed consent.

Clinicopathologic parameters were retrospectively collected from the medical records and are presented in Table 1. Briefly, in this cohort of 105 individuals with a median age of 65 years, there is a predominance of male patients (77.1%) and grade 2 HCC (74.3%), while the extent of the primary tumor is mostly pT2 (56.2%) and pT1 (36.2%).

### 2.2. Sample Preparation and Segmentation

**Figure 2:**
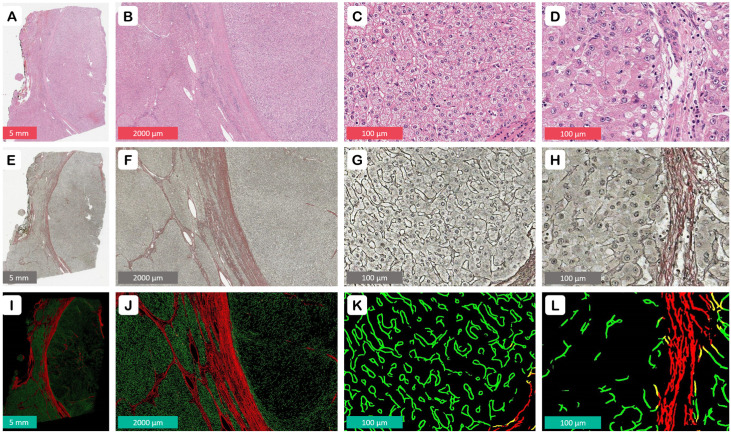
Staining and segmentation: (**A**–**D**) hematoxylin–eosin (H&E) staining is inferior to GSPS in the frame of this study due to its limited capacity to highlight the fibrous structures, such as the tumor capsule and septation of cirrhotic liver parenchyma; (**E**–**H**) a modified Gordon and Sweet’s silver impregnation protocol combined with Picric Acid–Sirius Red (GSPS). The nodularity of the cirrhotic liver and the encapsulation of the tumor are highlighted by red-stained bands of collagen (**F**); note the regular black linear reticulin network of the liver (**G**) in contrast to the loss of reticulin in HCC (**H**); (**I**–**L**) the CNN-generated pixel-precise mask of red (collagen) and green (reticulin) fibers set against a contrasting black background, optimized for viewing and morphometric feature extraction.

A pathologist (RS) reviewed all archived slides to identify the most representative formalin-fixed paraffin-embedded (FFPE) block. The selected block includes non-necrotic HCC tissue, and, whenever possible (in 103 samples, accounting for 98.1% of the cases), the surrounding peritumoral liver parenchyma. The 3 µm sections were stained using a modified Gordon and Sweet’s silver impregnation protocol combined with Picric Acid–Sirius Red, referred to as GSPS (see Figure 2E–H, supplementary Table S1). This method is standard for liver and bone marrow samples at the National Center of Pathology. Throughout this paper, we define the red-staining fibers in the thick fibrous septae as ‘collagen’, and the delicate black linear strands mostly located in the epithelial areas as ‘reticulin’.

**Figure 3:**
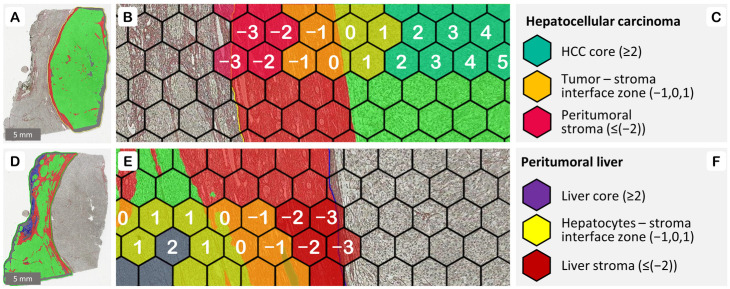
Hexagonal tiling, ranking, and zones of interest: (**A**) HALO^®^ AI (Indica Labs, Albuquerque, NM, USA) classifier result on the manually placed HCC annotation; (**B**) ranking of the hexagonal tiles according to their shortest distance from the tumor edge (rank 0 hexagon); (**C**) regions of interest within the HCC annotation; (**D**) classifier result for the manually annotated non-malignant liver parenchyma; (**E**) ranking of the hexagons on the sides of non-malignant epithelial edge; (**F**) regions of interest within the peritumoral liver annotation.

All slides were subsequently digitized at 20× magnification (0.5 μm per pixel) using an Aperio^®^ AT2 DX scanner (Leica Aperio Technologies, Vista, CA, USA). A pathologist (RS) reviewed the images to mark the malignant (HCC) and non-malignant (peritumoral liver) areas on each slide by placing manual annotations (see Figure 3A,D). A HALO^®^ AI (Indica Labs, Albuquerque, NM, USA) classifier was used to categorize the tissue into hepatocytes (indiscriminately malignant and non-malignant), stroma, and background classes.

### 2.3. CNN-Based Fiber Framework Extraction

We used a modified version of a U-Net model architecture proposed by Morkunas et al. in 2021 [28] to analyze the images of GSPS-stained samples. The model was designed to produce three output channels, representing collagen (red), reticulin (green), and an empty channel (black). This produces an RGB image, which is easy to view and analyze (see Figure 2I–L).

To produce the ground truth for model training, we selected 85 large (2048 × 2048 pixel-sized) regions of interest (ROIs) in 31 whole slide images (WSIs). We manually annotated fibrous structures of both collagen and reticulin in all ROIs by freeform annotation to produce RGB annotation masks of the fibers. Each region of interest (ROI) and its corresponding mask was then partitioned into 256 × 256-pixel image patches to match the model’s input size, resulting in a total of 5440 patches (N = 85 × (2048/256)^2^). To increase the training set, we augmented the dataset with 90° rotations, vertical and horizontal flips, blurring, zooming, and annotation erosion or dilation. Examples of annotated image patches are shown in Supplementary Figure S1. The final augmented dataset contained 54,400 image patches. The dataset was randomly divided into an 80% training subset and a 20% validation subset for model training.

During inference, the target WSI is divided into overlapping patches of size 256 × 256 pixels, with a step-size of 128 pixels in both vertical and horizontal directions on the image plane. Pixels in both red and green layers of the predicted fiber mask undergo separate thresholding processes. A pixel in the predicted collagen mask is assigned a value of 1.0 if the probability of collagen detection in that pixel exceeds 0.5; otherwise, it is assigned a value of 0.0. No rules were implemented to prevent a single target pixel from being attributed to more than one output layer. This lack of constraint allows for model uncertainty, manifesting as yellow pixels in the predicted RGB images where red and green fibers overlap, despite the absence of overlapping red and green fiber annotations in the ground truth.

The model was trained with adaptive moment estimation optimizer (using default parameters provided in the original method [31]) minimizing the binary cross-entropy loss function. We trained the model on single patch batches. Model weights were saved after each improvement in validation loss. The training phase was terminated after validation loss did not improve for 20 consecutive epochs. We used a suite of software tools including h5py (2013, Collete A., Boulder, CO, USA), numpy (version 1.20.0), and tensorflow with tensorboard (version 2.7.0).

### 2.4. Hexagonal Grid Tiling

Grid subsampling was utilized to sample the whole slide images (WSI) into hexagonal tiles (in this study having a long diagonal of 780 pixels) and rank them according to the distance to the automatically detected edge between the epithelium and stroma tissue classes, as described previously [30], see Figure 3. The tiling offers a number of advantages over processing the entire slide at once, with two of them being crucial. First, tiles allow a localized analysis of the tumor features, thus helping us identify the gradual changes and heterogeneity within different regions of the tissue, which might be overlooked in a whole-image analysis. Secondly, the spatially ranked tiles enable the extraction of specific regions of interest within the tumor, thereby providing more targeted insights. Briefly, during the ranking step, the hexagons at the stroma–epithelium (either benign hepatocytes or neoplastic HCC cells) boundary are identified and assigned a rank 0. Subsequently, the epithelial-side hexagons are attributed a positive rank, and stromal hexagons a negative rank, according to their shortest distance from the edge (rank 0 hexagon).

### 2.5. Calculation of Fiber Texture Descriptors

Morkunas et al. [28] originally proposed a set of 37 pixel-level, fiber-level, and image-level features to describe the CNN-derived fibrous matrix of breast cancer tissue. Drawing on past experience, we have reduced the number of features by removing the ones that were previously found to be the most correlated, dependent upon tissue placement on the glass slide, or difficult to interpret. In this study, for each tissue type marked by a pathologist, we calculated 11 features (see Table 2) including fiber orientation, morphometrics, fractal characteristics, and Haralick’s texture descriptors. Since these features are calculated per channel, separate descriptor sets were obtained for the green (reticulin) and the red (collagen) fibers, and the final number of calculated tissue characteristics is 22. Features were extracted for each hexagon, thus enabling analysis of fiber features according to hexagonal rank.

### 2.6. Statistical Analysis

The main goal of this study was to determine the impact of reticulin and collagen patterns in the tumor microenvironment on overall survival. Both the HCC and the peritumoral liver areas (as determined by a pathologist’s annotation), were further subdivided into three regions of interest each (see Figure 3): the three hexagon-wide ‘interface zone’ (IZ3), consisting of ranks [−1, 0, 1]; the epithelial ‘core’, incorporating ranks [≥2]; and the ‘stroma’, with the remaining ranks [≤(−2)]. For survival analysis, the data from individual hexagons across each of the six regions had to be aggregated on a per-case basis. During this aggregation, the mean and standard deviation of every feature measurement (across all hexagons, in each region of interest) are calculated for every case. The compiled case-level dataset contains 264 potential fiber-derived predictors, representing 2 annotations × 3 regions per annotation × 2 summary metrics (mean and standard deviation) × 2 types of fibers (green and red) × 11 fiber features. Twelve clinicopathological variables such as patient age, gender, tumor grade, size, stage, intravascular invasion, etc. were added to the set.

The data were analyzed using SAS software (version 9.4; SAS Institute Inc., Cary, NC, USA) and Python libraries (Pandas version 1.3.4, Scikit-learn version 1.0.2 and Lifelines version 0.27.0). Descriptive statistics were computed to summarize the main features of the data, with means, medians and ranges reported for continuous variables, and frequencies and percentages for categorical variables. Min–max scaling (or min–max normalization) was used to transform the metrics of the features to be on a similar scale in a fixed range between 0 and 1. Univariate Cox regression analysis with LASSO (least absolute shrinkage and selection operator) regularization was used to assess the performance of individual variables. Features with a *p* < 0.1 were used to construct the survival prediction models that underwent further evaluation using multivariate LASSO Cox analysis. After running LASSO Cox regression, only the models where all variables had *p* < 0.05 were retained. The C-index on the five-fold cross-validation test set was used to measure performance and rank the models accordingly. The individual features included in these models were ranked based on the frequency of their combined occurrence across all the models. Given the extensive list of significant predictors, we used the factor analysis as an additional step to identify underlying relationships among the variables, aiding interpretation.

## 3. Results

### 3.1. Descriptive Statistics

A total of 162,435 hexagonal tiles comprised the complete hexagon-level dataset. The range of measurements of reticulin and collagen fiber features in the individual hexagons prior to min–max scaling is presented in Table 3.

### 3.2. Univariate Predictors of Overall Survival

After scaling to standardize feature metrics, the impact of individual variables on the overall survival (OS) was evaluated using univariate Cox regression with LASSO regularization. Fifteen variables were determined to be statistically significant univariate predictors of OS. Higher stage (pT2-4, *p* = 0.0026), older patient age at the time of diagnosis (≥55 years, *p* = 0.0074), and the presence of intravascular invasion (*p* = 0.0089) were the strongest predictors of shorter OS in this cohort. Twelve fiber-derived features, all derived from the HCC ‘interface zone’ or ‘core’ regions, had *p* < 0.05, while showing either a positive or negative effect on OS. For a more inclusive approach, allowing for a comprehensive assessment in the subsequent multivariate Cox regression analysis, 36 features with *p* < 0.1 were selected for further modelling (see Table 4). The expanded list included multiple tumor nodules in the resected liver, alongside the addition of fiber-derived features from the peritumoral stroma, and the non-neoplastic liver parenchyma.

### 3.3. Multivariate Analysis

**Figure 4:**
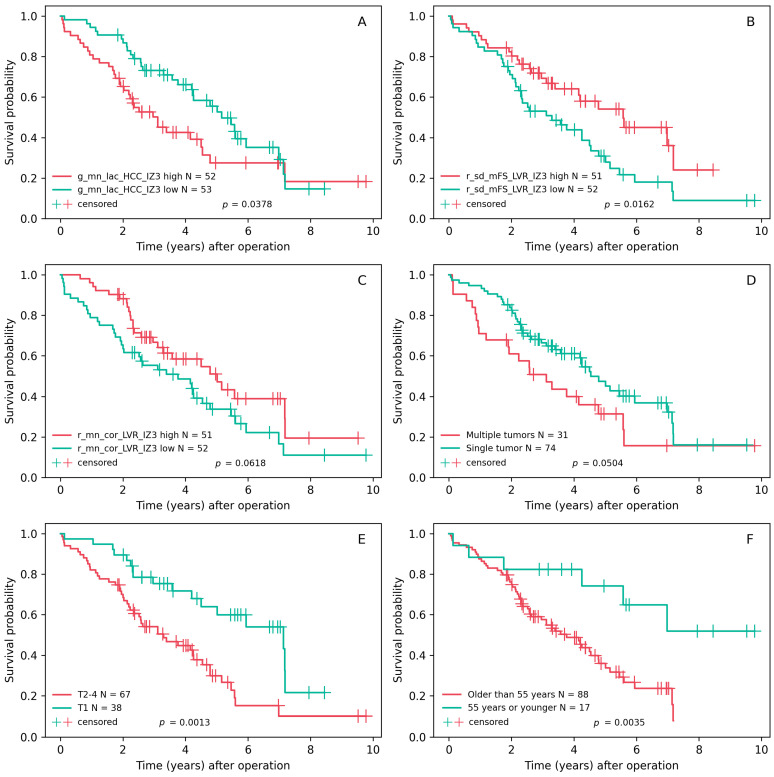
(**A**–**F**) Kaplan–Meier overall survival plots and the cutoff values for the components of models with good discriminative ability (C-index > 0.7); the best univariate predictor is also added.

In adherence with the ‘rule of ten’ guideline, which suggests at least 10 events per variable in constructing a regression model, and given the presence of 56 events in our dataset, we systematically generated and listed all the possible regression models (N = 2,835,199) containing 1, 2, 3, 4, 5 or 6 components from the previously defined set of features. After applying LASSO Cox regression, only those models (N = 139) in which all variables exhibited a *p*-value of less than 0.05 were retained (the complete list is presented in the Supplementary Table S2). The concordance index (C-index) on a five-fold cross-validation test set was chosen as the metric of performance, and the models were ranked accordingly. A C-index above 0.7 indicates good discriminative ability, and two similar models in our study have shown values above this threshold (see Table 5). Both models include a demographic variable (patient age), a pathological parameter (HCC multifocality), a reticulin-derived feature at the tumor edge, and the collagen-derived feature at the epithelial edge of peritumoral liver. The Kaplan–Meier OS plots (the cutoff values for groups here are the median values) for the individual components of these two models are presented in Figure 4.

The models in which every component had a *p*-value less than 0.05, were further analyzed by counting the number of times each variable appeared. Of the 139 models considered, all were composed of just 30 unique components in different combinations. The variables (features) were ranked based on the number of occurrences, and are presented in Table 6.

### 3.4. Factor Analysis

**Figure 5:**
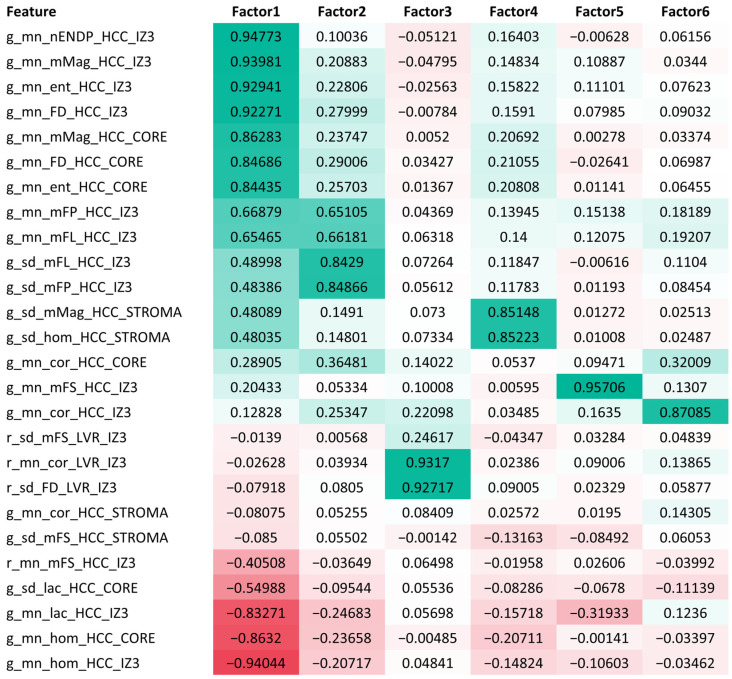
The heatmap of factor loadings (sorted by the loadings in Factor 1). Positive loadings in dark green boxes, negative loadings in red. Color intensity indicates magnitude, with dark green for high positive loadings and light green for low positive loadings.

We have further applied a factor analysis to investigate the underlying relationships of the fiber-derived features (N = 26) after removing the conventional clinicopathologic variables from the list of significant predictors. Six factors explaining 85.12% of the variance in the data were kept, using an eigenvalue of 1 as the criterion. Orthogonal varimax rotation was used to maximize the variance of the loadings within factors (the results are presented in Figure 5).

Factor 1 explains 53.73% of data variance and is characterized by the irregularity of reticulin fibers within the tumor–stroma interface and, to the lesser extent, the tumor core. Factor 1 has high loadings for variables like density, entropy, magnitude, and the number of endpoints of the reticulin fibers. Conversely, it displays negative loadings for parameters such as lacunarity and homogeneity. Factor 2 covers 11.08% of variance and highlights the heterogeneity of the reticulin fiber measurements, also within the same tumor–stroma interface. It is primarily influenced by variables corresponding to reticulin fiber length and fiber path, with emphasis on both mean values and standard deviations (SD) at the tumor edge. Factor 3 (6.56% of variance) captures the irregularity of the collagen network within the peritumoral liver–stroma interface, representing fibrosis around the functioning hepatocytes. It is mostly associated with the standard deviation of red pixel density per hexagon, and mean texture correlation of the collagen fibers.

Each of the other three factors covers less than 5% of the data variance. Factor 4 represents the heterogeneity of reticulin fibers in the peritumoral stroma, particularly in the standard deviation of homogeneity and magnitude. The pronounced straightness of reticulin fibers in the tumor–stroma interface forms the most of Factor 5. Lastly, Factor 6 emphasizes the correlation of reticulin fibers in all of the HCC tissue, represented by both the tumor–stroma interface, and the tumor core.

The first five factors exhibited cutoff values that divided the cohort into groups with statistically significant differences in overall survival duration, as indicated by a *p*-value of less than 0.05 in univariate analysis. However, after applying the LASSO Cox regression, none of these factors were found to be significant in the models, and therefore, they did not outperform the individual features.

## 4. Discussion

This study demonstrates the prognostic value of convolutional neural network-based mapping of reticulin and collagen fiber architecture in the HCC microenvironment. The integration of computational features describing the reticulin and collagen texture with the clinical parameters resulted in two multivariate overall survival models with a test cohort C-index > 0.7 after penalized LASSO Cox regression. Both models reveal the independent prognostic impact of patient age, tumor multifocality and fiber-derived features at the interfaces of HCC and the remaining functional hepatocytes with the surrounding stroma. Also, the reticulin structure provided the prognostic value at the tumor edge, while at the border of liver parenchyma, the collagen structure was relevant. Meanwhile, none of the models consisting of conventional clinicopathologic metrics only were able to surpass the 0.7 C-index threshold.

We have discovered that among the HCC-derived features in our cohort, the mean lacunarity of the reticulin framework at the tumor margin was the best-performing metric. Following closely, as indicated by its recurrent appearances in the models (Table 6), is the variance (SD) of the reticulin lacunarity at the core of the tumor. This observation aligns with the established significance of reticulin which is a key element in the structure of a normal liver, providing the framework of its lobular architecture. The alteration, disruption or dissipation of reticulin fibers is a well-documented diagnostic sign of HCC, first reported nearly 50 years ago [32]. Lacunarity is a fractal parameter that captures both gaps (Lat. lacuna) and heterogeneity in a pattern. In the context of our study, higher lacunarity would suggest the reduction and distortion of the reticulin framework, possibly indicating a more aggressive tumor phenotype. The high variance of lacunarity at the central part of the tumor—the core—might suggest the existence of areas with different degrees of aggressiveness. This insight finds parallels with a recent study by Patil A. et al., which confirmed a reduction in the AI-identified reticulin proportionate area (RPA) in HCC as a strong predictor of adverse patient outcomes [25]. However, our work revealed a potential superiority of spatial variations in the reticulin framework, as captured by lacunarity. The underperformance of the mean fiber density (FD, derived from the number of pixels in the mask and comparable to RPA) in our study, in contrast to lacunarity, suggests that the spatial heterogeneity in reticulin arrangement may provide more insight than merely the proportion of reticulin in the HCC tissue.

In cancer diagnostics, the non-neoplastic component of the tissue often receives somewhat lesser attention. However, our findings also underscore the independent prognostic significance of the peritumoral liver parenchyma. The extent of fibrosis, a well-documented predictor of chronic liver disease outcomes, can be assessed using a variety of invasive or non-invasive methods [33,34]. We have demonstrated that collagen in the peritumoral liver serves as a significant source of prognostic information, in contrast to the role of reticulin in HCC, as previously discussed. This aligns with the known role of Type I collagen as the primary component of fibrous tissue that accumulates during persistent liver damage. In our study, two features associated with peritumoral liver collagen were included in the most predictive models of overall survival (Table 5): the mean texture correlation of collagen fibers, and the high variance of collagen fiber straightness. Additionally, the high variance in fiber density emerged as the third collagen-derived feature, listed among the ten most recurrent components in the prognostic models (Table 6). Importantly, all these indicators were measured at the interface between remaining functional hepatocytes and the fibrous tissue (hexagon ranks −1, 0, 1). The standard deviation of individual measurements serves as an indicator of variability or heterogeneity between hexagons. In this case, it can highlight regions of dense, compact fibrosis in contrast to areas of the liver that remain somewhat intact, characterized by sparsely deposited and less organized collagen fibers. On the other hand, a more consistent overall tissue structure (reflected by the high mean correlation), might indicate a less advanced liver disease with higher residual functional capacity. Combined, the high variability of collagen deposition in the individual hexagons and the maintenance of general parenchymal integrity might indicate the presence of ongoing successful tissue repair. As sufficient residual liver function is crucial for the survival of patients [35], its assessment alongside tumor parameters on the same tissue slide offers a streamlined and practical approach in HCC resection samples.

A factor analysis was used to investigate the inherent relations between the 26 fiber-derived features that, when combined in certain ways (see Supplementary Table S2), showed statistically significant predictive power (*p* < 0.05) in regression models. Six factors were identified, capturing the majority of the information in the original data. The heatmap in Figure 5 highlights the strong negative association between the mean reticulin lacunarity at the tumor edge (g_mn_lac_HCC_IZ3) and the dominant Factor 1, positioning it as a key determinant. Notably, this variable also exhibits the most negative loadings for both Factor 2 and Factor 5, and ranks second to last in terms of negative loadings for Factor 4. These consistent negative loadings across four of the six factors suggest that g_mn_lac_HCC_IZ3 captures multidimensional information about the reticulin framework at the tumor edge, making it the most prominent HCC-derived feature. Furthermore, the predominant features defining peritumoral liver-derived Factor 3 reflect the consistency in collagen fiber orientation (r_mn_cor_LVR_IZ3), variability in density (r_sd_FD_LVR_IZ3), and variability in fiber straightness (r_sd_mFS_LVR_IZ3). Consequently, the pairing of a tumor-based g_mn_lac_HCC_IZ3 indicator with either r_mn_cor_LVR_IZ3 or r_sd_mFS_LVR_IZ3, both liver-based features, collectively covers Factors 1–5, which represent 81.04% of the variance. This unique combination demonstrates remarkable performance in Cox regression models and outperforms the factor values, emphasizing the synergistic role of tumor and liver characteristics in HCC prognostication.

Our study contains some limitations. The lack of complete data on HBV and HCV infections restricts a thorough examination of their potential impact on the overall survival in our HCC patients. Secondly, the lack of the information on the cause of death limits our options for predicting disease-specific survival, which would be relevant for our focus on impact of malignant and non-malignant components. Thirdly, the cohort of 105 patients is rather limited and serves as a proof-of-concept study. Validation studies on independent cohorts would be needed to assess generalizability of our findings.

## 5. Conclusions

We have focused on using a convolutional neural network to segment the two types of fibers from the histopathology images containing HCC and the adjacent liver tissue, which were then used to calculate the predictive biomarkers of overall survival. These biomarkers are based on the structure of reticulin and collagen fibers both in the tumor microenvironment and the adjacent residual non-neoplastic liver tissue. We underscore the critical importance of precise tissue zoning due to the concentration of prognostic information at the edges of both benign and malignant epithelial tissue. The dual-type fiber extraction method allowed us to confirm the central role of reticulin in HCC, while collagen emerged as a more predictive component in the peritumoral liver. Moreover, our findings show that fiber-derived features provide independent prognostic value, augmenting conventional clinicopathologic parameters such as patient age, tumor multifocality, intravascular invasion, and pT stage.

## Figures and Tables

**Table 1:** Patient and tumor characteristics of the study cohort.

Characteristic	Value (Range or Percent)
Patients	105 (100%)
Age, years	
Mean (range)	63.7 (13–82)
Median	65
≥55 years	88 (83.8)
Gender	
Male	81 (77.1%)
Female	24 (22.9%)
Metavir fibrosis stage	
F0 (no fibrosis)	12 (11.4%)
F1 (portal fibrosis without septa)	5 (4.8%)
F2 (portal fibrosis with rare septa)	11 (10.4%)
F3 (numerous septa without cirrhosis)	16 (15.3%)
F4 (cirrhosis)	61 (58.1%)
Largest tumor dimension, cm	
Mean (range)	4.76 (0.8–19.0)
Median	40
Tumor multinodularity	
Single HCC nodule	74 (70.5%)
Multiple HCC nodules	31 (29.5%)
HCC grade	
G1	8 (7.6%)
G2	78 (74.3%)
G3	19 (18.1%)
pT stage	
T1	38 (36.2%)
T2	59 (56.2%)
T3	7 (6.7%)
T4	1 (0.9%)
Intravascular invasion	
Present	52 (49.5%)
Absent	53 (50.5%)
Lymph nodes present	
Yes	19 (18.1%)
No	86 (81.9%)
Lymph nodes per patient if present, mean (median)	2.7 (2)
Metastatic spread confirmed	1/19 (5.3%)
Resection margin	
R0	85 (80.9%)
R1	20 (19.1%)
History of viral infection	
HBV	9 (8.6%)
HCV	52 (49.5%)
None or unknown	44 (41.9%)
Other treatment prior to current resection	
Yes	13 (12.4%)
No	92 (87.6%)
HCC recurrence after resection	
Yes	56 (53.3%)
No	49 (46.7%)
OS time, days	
Mean (range)	1108.7 (20–3160)
Median	938

**Table 2:** List of features used to characterize the fibrous matrix, calculated twice per each hexagon: for the green (reticulin or Type 3 collagen) and the red (Type 1 collagen) fibers separately.

Feature	Description
Orientation:	
Circular standard deviation (CSD)	Dispersion of circular angles of the individual fibers
Magnitude, mean (mMag)	Average strength or intensity of vectors (e.g., gradients) in the fiber mask
Morphometry:	
Fiber length, mean (mFL)	Average Euclidean distance between the endpoints of each skeletonized fiber
Fiber path, mean (mFP)	Average pixel length of a line dividing a fiber into two equal parts along its longer axis
Fiber straightness, mean (mFS)	A ratio of fiber length over fiber path
Density:	
Fiber density (FD)	Number of pixels in the mask
Endpoints (nENDP)	Number of fiber endpoints in the hexagon mask
Texture (Haralick’s):	
Homogeneity (hom)	The closeness of the distribution of elements in the gray-level co-occurrence matrix to the matrix diagonal
Entropy (ent)	Amount of information or randomness in the texture
Correlation (cor)	Linear dependency of gray levels on those of neighboring pixels
Fractal:	
Lacunarity (lac)	A measure of both gaps and heterogeneity: the variation in space around objects in the image and their irregular distribution

**Table 3:** Range of the reticulin (green, prefix ‘g_’) and collagen (red, prefix ‘r_’) fiber features as measured per individual hexagonal tiles prior to scaling.

Feature	Min	Max	Mean	Median
g_CSD	0.327599	1.551254	0.874630	0.872546
g_mMag	2.731371	25,599.785804	7410.622809	6869.993232
g_mFL	0.000000	1059.079906	61.729315	52.348669
g_mFP	2.500000	1246.933333	90.089874	78.200000
g_mFS	0.000000	0.956133	0.497333	0.507360
g_FD	1.000000	151,375.000000	34,755.695386	30,771.000000
g_nENDP	0.000000	1394.000000	322.604360	312.000000
g_hom	0.947687	0.999997	0.984834	0.985913
g_ent	0.000068	1.092885	0.380083	0.378187
g_cor	−0.000008	0.944431	0.843028	0.851353
g_lac	0.000000	1.206979	0.589991	0.579632
r_CSD	0.282147	1.583950	0.842409	0.824565
r_mMag	2.630596	34,292.920449	6742.703072	3761.257993
r_mFL	0.000000	28,507.246022	132.393359	51.126263
r_mFP	2.500000	14,861.500000	133.552675	72.366667
r_mFS	0.000000	3.478550	0.470136	0.482461
r_FD	1.000000	386,398.000000	45,713.265522	19,491.000000
r_nENDP	0.000000	2221.000000	360.981139	206.000000
r_hom	0.929537	0.999998	0.986157	0.992241
r_ent	0.000044	1.315616	0.380543	0.259431
r_cor	−0.000008	0.988216	0.839329	0.858877
r_lac	0.000000	1.295812	0.594719	0.602250

**Table 4:** Univariate predictors of overall survival ranked by the *p*-value (Cox regression with LASSO regularization).

Feature	*p*-Value	Hazard Ratio (HR)
Stage pT2-4	0.0026	2.3719
Age ≥ 55 years	0.0074	3.1990
Intravascular invasion	0.0089	1.9564
g_mn_mFL_HCC_IZ3	0.0095	0.0897
g_mn_mFP_HCC_IZ3	0.0095	0.1078
g_mn_cor_HCC_IZ3	0.0199	0.1114
g_sd_mFL_HCC_IZ3	0.0285	0.2203
g_mn_ent_HCC_IZ3	0.0294	0.2987
g_mn_FD_HCC_IZ3	0.0340	0.3049
g_mn_lac_HCC_IZ3	0.0343	4.1651
g_mn_mMag_HCC_IZ3	0.0356	0.3049
g_mn_hom_HCC_IZ3	0.0361	3.2656
g_mn_cor_HCC_CORE	0.0394	0.1385
g_sd_mFP_HCC_IZ3	0.0397	0.2500
g_mn_cor_HCC_STROMA	0.0482	0.1466
g_mn_mFS_HCC_IZ3	0.0514	0.1900
r_sd_mFS_LVR_IZ3	0.0532	0.0417
g_mn_nENDP_HCC_IZ3	0.0553	0.3200
g_mn_ent_HCC_CORE	0.0589	0.3644
g_sd_FD_HCC_STROMA	0.0595	0.3406
g_mn_mFP_HCC_STROMA	0.0702	0.1600
Multiple tumors	0.0717	1.6175
g_sd_mFS_HCC_STROMA	0.0733	2.6564
r_sd_FD_LVR_IZ3	0.0745	0.2762
g_mn_mFL_HCC_STROMA	0.0784	0.1616
r_mn_cor_LVR_IZ3	0.0858	0.2579
g_mn_FD_HCC_CORE	0.0865	0.3792
g_sd_mFS_HCC_IZ3	0.0902	3.0509
r_mn_mFS_HCC_IZ3	0.0935	4.2304
g_sd_ent_HCC_STROMA	0.0941	0.3704
g_sd_mMag_HCC_STROMA	0.0954	0.3911
g_sd_hom_HCC_STROMA	0.0963	0.3912
g_mn_hom_HCC_CORE	0.0986	2.5157
g_mn_mMag_HCC_CORE	0.0988	0.3972
g_mn_ent_HCC_STROMA	0.0995	0.2707
g_sd_lac_HCC_CORE	0.0995	3.1310

**Table 5:** Cox regression models with a C-index above 0.7.

Features	HR	95% CI	*p*-Value
Model A, test set C-index 0.7094, AIC 359.3840			
Age ≥ 55 years	4.05	1.67–9.80	0.00194
Multiple_tumors	1.92	1.11–3.31	0.01895
g_mn_lac_HCC_IZ3	6.36	1.69–23.87	0.00615
r_mn_cor_LVR_IZ3	0.21	0.05–0.92	0.03802
Model B, test set C-index 0.7061, AIC 359.2425			
Age ≥ 55 years	4.33	1.73–10.81	0.0017
Multiple_tumors	2.24	1.30–3.83	0.0035
g_mn_lac_HCC_IZ3	5.58	1.48–21.06	0.0113
r_sd_mFS_LVR_IZ3	0.02	0.00–0.84	0.0396

**Table 6:** List of the variables comprising statistically significant (*p* < 0.05) OS models ranked by the number of occurrences in the models.

Feature	Number of Occurrences
Age_55plus	57
r_mn_cor_LVR_IZ3	39
r_sd_mFS_LVR_IZ3	28
LVI	27
r_sd_FD_LVR_IZ3	23
Multiple_tumors	23
g_mn_lac_HCC_IZ3	16
g_sd_lac_HCC_CORE	13
pT2-3	12
g_mn_cor_HCC_IZ3	11
g_mn_mMag_HCC_IZ3	10
g_mn_hom_HCC_IZ3	10
g_mn_ent_HCC_IZ3	8
g_mn_FD_HCC_IZ3	7
g_mn_cor_HCC_STROMA	7
g_mn_mFL_HCC_IZ3	7
g_mn_ent_HCC_CORE	6
g_mn_mFP_HCC_IZ3	6
g_mn_hom_HCC_CORE	6
g_mn_mMag_HCC_CORE	6
g_mn_nENDP_HCC_IZ3	6
g_sd_mFS_HCC_STROMA	5
g_sd_mFL_HCC_IZ3	3
g_mn_mFS_HCC_IZ3	2
g_mn_FD_HCC_CORE	2
r_mn_mFS_HCC_IZ3	2
g_mn_cor_HCC_CORE	2
g_sd_mFP_HCC_IZ3	2
g_sd_mMag_HCC_STROMA	1
g_sd_hom_HCC_STROMA	1

## Data Availability

The datasets generated during the study are not publicly available due to permit restrictions. However, they can be made available for collaborative work upon reasonable request, subject to the approval of our team regarding the intended use of the data. Interested parties should reach out with specific collaboration proposals for consideration.

## Supplementary Materials

The following supporting information can be downloaded at: https://www.mdpi.com/article/10.3390/cancers16010106/s1, Table S1: GSPS staining protocol; Table S2: Models (N = 139) in which all variables exhibited a *p*-value of less than 0.05; Figure S1: Applied augmentations: rotation, flipping, blurring, zooming, and different amounts of annotation erosion and dilation applied to one image patch.
