# Supplementary material for: Improving HCC Prognostic Models after Liver Resection by AI-Extracted Tissue Fiber Framework Analytics
Source: Cancers (Basel). 2023 Dec 24;16(1):106. doi: 10.3390/cancers16010106 (PMC10778366; doi:10.3390/cancers16010106)
Supplement: Supplementary file 1 [file cancers-16-00106-s001.zip › cancers-2721267-supplementary.pdf]

Supplemental Table ST1. GSPS staining protocol.

	Reagent	Action, time
	Sample type required: Deparaffinized and rehydrated tissue section mounted on a positively charged slide	
1.	1% potassium permanganate solution	5 minutes
2.	Running water	Wash
3.	5% oxalic acid solution	Wash until tissue is colorless
4.	Running water	Wash
5.	2.5% iron alum solution	15 minutes
6.	Distilled water	3 changes
7.	Ammoniacal silver solution (*ex tempore*)	2 minutes
8.	Distilled water	3 changes
9.	15% formalin solution	1 minute
10.	Running water	Wash
11.	0.2% gold chloride solution	1 minute
12.	Running water	Wash
13.	2.5% sodium thiosulphate solution	5 minutes
14.	Running water	Wash
15.	Weigert's iron hematoxylin	1-2 seconds
16.	Running water	Wash
17.	Picric acid – Sirius Red solution	3 minutes
18.	Tap water	1 second
19.	Dehydration with 96% alcohol and isopropyl alcohol, followed by xylene clearing	
20.	Cover slip	

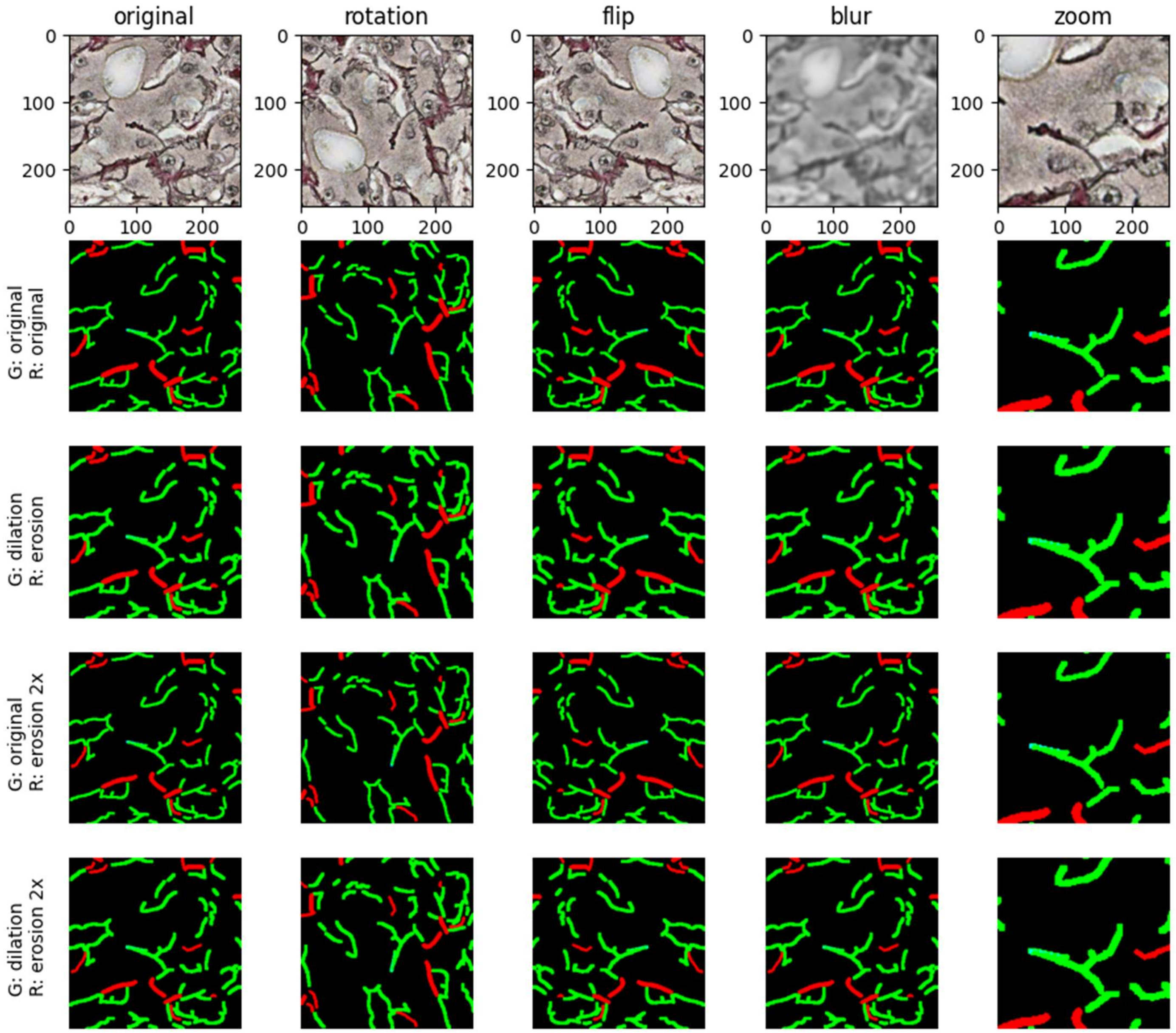

Supplemental Figure SF1. Applied augmentations - rotation, flipping, blurring, zooming, and different amount of annotation erosion and dilation applied to one image patch.

Supplemental Table ST2. Models (N=139) in which all variables exhibited a *p*-value of less than 0.05

Model	Mean training set C-index	Mean test set C-index	Mean AIC
Age_55plus + Multiple_tumors + g_mn_lac_HCC_IZ3 + r_mn_cor_LVR_IZ3	0.7064	0.7094	359.38
Age_55plus + Multiple_tumors + g_mn_lac_HCC_IZ3 + r_sd_mFS_LVR_IZ3	0.7122	0.7061	359.24
Age_55plus + Multiple_tumors + g_mn_mMag_HCC_IZ3 + r_sd_mFS_LVR_IZ3	0.6946	0.6915	360.78
Age_55plus + Multiple_tumors + g_mn_ent_HCC_IZ3 + r_sd_mFS_LVR_IZ3	0.6953	0.6911	360.39
Age_55plus + Multiple_tumors + g_mn_hom_HCC_IZ3 + r_sd_mFS_LVR_IZ3	0.6943	0.6903	360.80
Age_55plus + Multiple_tumors + g_mn_FD_HCC_IZ3 + r_sd_mFS_LVR_IZ3	0.6948	0.6903	360.33
Age_55plus + g_mn_lac_HCC_IZ3 + r_mn_cor_LVR_IZ3	0.6967	0.6881	361.81
Age_55plus + Multiple_tumors + r_sd_mFS_LVR_IZ3 + g_mn_lac_HCC_CORE	0.6987	0.6864	360.98
Age_55plus + Multiple_tumors + r_sd_mFS_LVR_IZ3 + g_mn_ent_HCC_CORE	0.6928	0.6826	360.77
Age_55plus + g_mn_lac_HCC_IZ3 + r_mn_cor_LVR_IZ3 + pT2-3	0.7150	0.6825	353.83
Age_55plus + Multiple_tumors + r_sd_mFS_LVR_IZ3 + g_mn_FD_HCC_CORE	0.6892	0.6812	361.05
Age_55plus + Multiple_tumors + g_mn_nENDP_HCC_IZ3 + r_mn_cor_LVR_IZ3	0.6877	0.6810	362.31
Age_55plus + Multiple_tumors + r_sd_mFS_LVR_IZ3 + g_mn_nENDP_HCC_CORE	0.6881	0.6807	361.11
Age_55plus + LVI + g_mn_lac_HCC_IZ3 + r_mn_cor_LVR_IZ3	0.7112	0.6804	356.39
Age_55plus + Multiple_tumors + r_sd_mFS_LVR_IZ3 + g_mn_mMag_HCC_CORE	0.6903	0.6784	361.03
Age_55plus + Multiple_tumors + r_sd_mFS_LVR_IZ3 + g_sd_lac_HCC_CORE	0.6993	0.6782	360.45
g_mn_lac_HCC_IZ3 + r_mn_cor_LVR_IZ3 + pT2-3	0.6953	0.6775	363.79
Age_55plus + Multiple_tumors + r_sd_mFS_LVR_IZ3 + g_mn_hom_HCC_CORE	0.6903	0.6772	361.03
Age_55plus + LVI + g_mn_lac_HCC_IZ3 + r_sd_FD_LVR_IZ3	0.7069	0.6771	357.78
Age_55plus + LVI + g_mn_mFL_HCC_IZ3 + r_mn_cor_LVR_IZ3	0.7031	0.6765	356.54
Age_55plus + LVI + Multiple_tumors + r_sd_mFS_LVR_IZ3 + g_sd_lac_HCC_CORE	0.7245	0.6763	355.82
Age_55plus + g_mn_lac_HCC_IZ3 + r_sd_FD_LVR_IZ3	0.6904	0.6739	362.44
Age_55plus + g_mn_hom_HCC_IZ3 + r_mn_cor_LVR_IZ3	0.6800	0.6730	363.80
Age_55plus + Multiple_tumors + r_sd_mFS_LVR_IZ3	0.6806	0.6719	362.41
Age_55plus + g_mn_mMag_HCC_IZ3 + r_mn_cor_LVR_IZ3	0.6803	0.6715	363.78
Age_55plus + r_sd_FD_LVR_IZ3 + g_mn_ent_HCC_CORE	0.6707	0.6702	364.56
Age_55plus + g_mn_ent_HCC_IZ3 + r_mn_cor_LVR_IZ3	0.6829	0.6690	363.40

Model	Mean training set C-index	Mean test set C-index	Mean AIC
Age_55plus + g_mn_mFP_HCC_IZ3 + r_mn_cor_LVR_IZ3	0.6764	0.6685	362.60
Age_55plus + g_mn_FD_HCC_IZ3 + r_sd_FD_LVR_IZ3	0.6739	0.6682	364.08
Age_55plus + LVI + r_mn_cor_LVR_IZ3 + g_sd_lac_HCC_CORE	0.6983	0.6674	356.79
Age_55plus + g_mn_FD_HCC_IZ3 + r_mn_cor_LVR_IZ3	0.6805	0.6665	363.62
g_mn_mFS_HCC_IZ3 + pT2-3	0.6727	0.6662	367.88
LVI + g_mn_lac_HCC_IZ3 + r_mn_cor_LVR_IZ3	0.6885	0.6660	366.29
Multiple_tumors + r_sd_mFS_LVR_IZ3 + g_mn_mMag_HCC_CORE	0.6702	0.6653	368.39
Multiple_tumors + r_sd_mFS_LVR_IZ3 + g_mn_hom_HCC_CORE	0.6703	0.6653	368.38
Age_55plus + LVI + r_mn_cor_LVR_IZ3	0.6854	0.6629	358.10
Age_55plus + g_mn_mFL_HCC_IZ3 + r_mn_cor_LVR_IZ3	0.6748	0.6629	362.58
g_mn_mMag_HCC_IZ3 + r_mn_cor_LVR_IZ3 + pT2-3	0.6877	0.6625	363.72
Multiple_tumors + r_sd_mFS_LVR_IZ3 + g_mn_nENDP_HCC_CORE	0.6656	0.6615	367.79
g_mn_nENDP_HCC_IZ3 + r_mn_cor_LVR_IZ3 + pT2-3	0.6850	0.6605	364.23
Age_55plus + Multiple_tumors + g_mn_lac_HCC_IZ3	0.6727	0.6599	368.79
g_mn_hom_HCC_IZ3 + r_mn_cor_LVR_IZ3 + pT2-3	0.6875	0.6597	363.75
Age_55plus + r_sd_mFS_LVR_IZ3 + g_mn_lac_HCC_CORE	0.6658	0.6594	364.38
Age_55plus + r_sd_FD_LVR_IZ3 + g_mn_lac_HCC_CORE	0.6683	0.6594	364.57
Age_55plus + g_mn_ent_HCC_IZ3 + r_sd_FD_LVR_IZ3	0.6742	0.6593	363.84
Age_55plus + g_mn_mMag_HCC_IZ3 + r_sd_FD_LVR_IZ3	0.6712	0.6586	364.29
Age_55plus + g_mn_hom_HCC_IZ3 + r_sd_FD_LVR_IZ3	0.6707	0.6586	364.30
Age_55plus + LVI + g_mn_cor_HCC_IZ3	0.6724	0.6576	363.80
Age_55plus + Multiple_tumors + g_mn_mFL_HCC_IZ3	0.6746	0.6571	367.40
g_mn_mFP_HCC_IZ3 + pT2-3	0.6659	0.6554	367.45
Age_55plus + Multiple_tumors + g_mn_mFP_HCC_IZ3	0.6713	0.6544	367.58
g_mn_mFL_HCC_IZ3 + pT2-3	0.6669	0.6543	367.23
Multiple_tumors + r_sd_mFS_LVR_IZ3 + g_sd_lac_HCC_CORE	0.6745	0.6542	367.74
Age_55plus + r_sd_mFS_LVR_IZ3 + g_mn_FD_HCC_CORE	0.6596	0.6539	364.93
Multiple_tumors + r_sd_mFS_LVR_IZ3	0.6527	0.6536	369.72
LVI + g_mn_mFS_HCC_IZ3	0.6687	0.6521	370.79
LVI + g_mn_mFP_HCC_IZ3	0.6634	0.6519	370.29
Age_55plus + r_sd_mFS_LVR_IZ3 + g_mn_ent_HCC_CORE	0.6604	0.6517	364.63
LVI + g_mn_cor_HCC_IZ3 + g_sd_mFS_HCC_STROMA	0.6828	0.6513	367.51
Age_55plus + LVI + r_sd_FD_LVR_IZ3 + g_sd_lac_HCC_CORE	0.6929	0.6509	357.51
LVI + g_mn_mFL_HCC_IZ3	0.6646	0.6506	370.12
Age_55plus + r_mn_cor_LVR_IZ3 + g_sd_lac_HCC_CORE	0.6638	0.6503	363.78
Age_55plus + g_mn_cor_HCC_IZ3 + pT2-3	0.6596	0.6499	361.83
Age_55plus + r_sd_mFS_LVR_IZ3 + g_mn_hom_HCC_CORE	0.6602	0.6498	364.91
Age_55plus + r_sd_mFS_LVR_IZ3 + g_mn_mMag_HCC_CORE	0.6599	0.6498	364.91
LVI + g_mn_mMag_HCC_IZ3 + r_mn_cor_LVR_IZ3	0.6754	0.6489	365.70
LVI + g_mn_hom_HCC_IZ3 + r_mn_cor_LVR_IZ3	0.6754	0.6489	365.72

Model	Mean training set C-index	Mean test set C-index	Mean AIC
LVI + r_mn_cor_LVR_IZ3 + g_mn_nENDP_HCC_CORE	0.6692	0.6488	366.42
LVI + g_mn_hom_HCC_IZ3 + r_sd_FD_LVR_IZ3	0.6745	0.6483	366.24
LVI + g_mn_mMag_HCC_IZ3 + r_sd_FD_LVR_IZ3	0.6745	0.6483	366.22
LVI + g_mn_ent_HCC_IZ3 + r_mn_cor_LVR_IZ3	0.6749	0.6483	365.54
LVI + r_mn_cor_LVR_IZ3 + g_sd_lac_HCC_CORE	0.6694	0.6483	366.16
Age_55plus + r_sd_mFS_LVR_IZ3 + g_sd_lac_HCC_CORE	0.6578	0.6464	363.67
Age_55plus + r_sd_FD_LVR_IZ3	0.6291	0.6457	365.87
Age_55plus + g_sd_mFL_HCC_IZ3 + r_mn_cor_LVR_IZ3	0.6667	0.6457	363.75
Age_55plus + g_mn_lac_HCC_IZ3	0.6424	0.6443	369.82
LVI + g_mn_nENDP_HCC_IZ3 + r_sd_FD_LVR_IZ3	0.6690	0.6439	366.65
LVI + g_mn_nENDP_HCC_IZ3 + r_mn_cor_LVR_IZ3	0.6694	0.6431	366.13
g_mn_cor_HCC_IZ3 + pT2-3	0.6375	0.6424	367.80
LVI + g_mn_FD_HCC_IZ3 + r_mn_cor_LVR_IZ3	0.6730	0.6414	365.62
Age_55plus + r_sd_FD_LVR_IZ3 + g_sd_lac_HCC_CORE	0.6553	0.6403	363.76
LVI + g_mn_cor_HCC_IZ3	0.6494	0.6393	369.58
g_mn_lac_HCC_IZ3 + g_mn_cor_HCC_STROMA	0.6436	0.6391	373.14
g_mn_lac_HCC_IZ3 + r_mn_cor_LVR_IZ3	0.6559	0.6379	369.45
Age_55plus + r_sd_ent_LVR_IZ3 + g_sd_lac_HCC_CORE	0.6545	0.6379	365.10
g_mn_lac_HCC_IZ3 + r_sd_FD_LVR_IZ3	0.6563	0.6379	369.64
g_mn_mMag_HCC_IZ3 + r_sd_FD_LVR_IZ3	0.6368	0.6377	369.85
g_mn_hom_HCC_IZ3 + r_sd_FD_LVR_IZ3	0.6367	0.6377	369.87
g_mn_ent_HCC_IZ3 + r_sd_FD_LVR_IZ3	0.6405	0.6374	369.45
Age_55plus + g_mn_mFL_HCC_IZ3	0.6378	0.6365	368.59
Age_55plus + g_mn_mFP_HCC_IZ3	0.6358	0.6350	368.72
LVI + g_mn_cor_HCC_STROMA	0.6334	0.6342	371.47
r_mn_cor_LVR_IZ3 + g_mn_nENDP_HCC_CORE	0.6324	0.6306	370.72
r_mn_mFS_HCC_IZ3 + r_mn_cor_LVR_IZ3	0.6408	0.6305	371.47
Age_55plus + r_mn_cor_LVR_IZ3	0.6417	0.6299	365.42
r_mn_cor_LVR_IZ3 + g_mn_ent_HCC_CORE	0.6326	0.6285	370.55
r_mn_cor_LVR_IZ3 + g_mn_mMag_HCC_CORE	0.6331	0.6283	370.94
g_mn_cor_HCC_IZ3 + g_mn_lac_HCC_IZ3	0.6401	0.6276	372.48
r_sd_mFS_LVR_IZ3 + g_sd_lac_HCC_CORE	0.6301	0.6274	369.44
Age_55plus + LVI	0.6275	0.6274	366.11
r_mn_cor_LVR_IZ3 + g_mn_hom_HCC_CORE	0.6333	0.6269	370.92
r_sd_FD_LVR_IZ3 + g_mn_mMag_HCC_CORE	0.6238	0.6262	370.95
r_sd_FD_LVR_IZ3 + g_mn_hom_HCC_CORE	0.6243	0.6262	370.94
g_mn_FD_HCC_IZ3 + r_mn_cor_LVR_IZ3	0.6414	0.6255	369.52
r_sd_FD_LVR_IZ3 + g_mn_nENDP_HCC_CORE	0.6241	0.6255	370.71
g_mn_cor_HCC_CORE + g_sd_mFS_HCC_STROMA	0.6233	0.6254	373.30
LVI + r_sd_FD_LVR_IZ3 + g_sd_lac_HCC_CORE	0.6666	0.6253	366.11
Age_55plus + Multiple_tumors + g_mn_cor_HCC_IZ3	0.6464	0.6250	367.50
g_mn_nENDP_HCC_IZ3 + r_sd_FD_LVR_IZ3	0.6321	0.6248	370.53
g_mn_mMag_HCC_IZ3 + r_mn_cor_LVR_IZ3	0.6452	0.6216	369.68
r_sd_mFS_LVR_IZ3 + g_mn_hom_HCC_CORE	0.6310	0.6216	370.52
r_sd_mFS_LVR_IZ3 + g_mn_mMag_HCC_CORE	0.6312	0.6216	370.52
r_sd_mFS_LVR_IZ3 + g_mn_ent_HCC_CORE	0.6328	0.6213	370.13

Model	Mean training set C-index	Mean test set C-index	Mean AIC
r_sd_FD_LVR_IZ3 + g_mn_ent_HCC_CORE	0.6276	0.6203	370.55
g_mn_hom_HCC_IZ3 + r_mn_cor_LVR_IZ3	0.6454	0.6201	369.71
r_sd_mFS_LVR_IZ3 + g_sd_mMag_HCC_STROMA	0.6191	0.6201	370.57
g_mn_ent_HCC_IZ3 + r_mn_cor_LVR_IZ3	0.6439	0.6198	369.31
g_mn_lac_HCC_IZ3	0.6180	0.6197	374.85
r_sd_mFS_LVR_IZ3 + g_sd_hom_HCC_STROMA	0.6187	0.6189	370.58
r_sd_mFS_LVR_IZ3 + g_mn_nENDP_HCC_CORE	0.6305	0.6187	370.47
g_sd_mFL_HCC_IZ3 + g_sd_mFS_HCC_STROMA	0.6356	0.6186	372.36
g_sd_mFP_HCC_IZ3 + g_sd_mFS_HCC_STROMA	0.6334	0.6184	373.01
g_mn_mFL_HCC_IZ3	0.6221	0.6168	372.18
g_mn_mFP_HCC_IZ3	0.6218	0.6168	372.32
Age_55plus + pT2-3	0.6294	0.6166	363.46
Age_55plus + g_mn_cor_HCC_IZ3	0.6198	0.6141	368.84
r_mn_cor_LVR_IZ3 + g_sd_lac_HCC_CORE	0.6093	0.6123	370.55
g_mn_nENDP_HCC_IZ3 + r_mn_cor_LVR_IZ3	0.6397	0.6114	370.47
g_mn_nENDP_HCC_CORE + g_mn_mFS_HCC_STROMA	0.6148	0.6107	374.84
g_mn_ent_HCC_IZ3 + g_mn_cor_HCC_STROMA	0.6231	0.6089	373.43
r_mn_mFS_HCC_IZ3 + r_sd_mFS_LVR_IZ3	0.6150	0.6067	371.00
pT2-3	0.6038	0.6057	370.37
LVI	0.6057	0.6048	372.76
g_mn_hom_HCC_IZ3 + g_mn_cor_HCC_STROMA	0.6173	0.6020	373.93
g_mn_mMag_HCC_IZ3 + g_mn_cor_HCC_STROMA	0.6174	0.6020	373.91
g_mn_FD_HCC_IZ3 + g_mn_cor_HCC_STROMA	0.6198	0.6016	373.54
r_sd_FD_LVR_IZ3 + g_sd_lac_HCC_CORE	0.6047	0.6005	370.12
g_mn_FD_HCC_IZ3	0.6019	0.5948	374.70
g_mn_ent_HCC_IZ3	0.6008	0.5896	374.72
g_mn_cor_HCC_IZ3 + g_sd_mFS_HCC_STROMA	0.6192	0.5822	372.26
Multiple_tumors + g_mn_cor_HCC_IZ3	0.6042	0.5813	372.54
g_sd_mFL_HCC_IZ3	0.5900	0.5805	374.00
g_mn_mMag_HCC_IZ3	0.5962	0.5802	375.36
g_mn_hom_HCC_IZ3	0.5962	0.5802	375.38
g_mn_cor_HCC_IZ3	0.5685	0.5740	373.79
g_sd_mFP_HCC_IZ3	0.5844	0.5714	374.60
g_mn_cor_HCC_STROMA	0.5663	0.5672	375.55
g_mn_cor_HCC_CORE	0.5579	0.5656	374.91
Age_55plus	0.5560	0.5540	371.02

7
